# Working memory in intact modalities among individuals with sensory deprivation

**DOI:** 10.1016/j.heliyon.2022.e09558

**Published:** 2022-05-29

**Authors:** Eyal Heled, Maayan Ohayon, Or Oshri

**Affiliations:** a Department of Psychology, Ariel University, Israel; b Department of Neurological Rehabilitation, Sheba Medical Center, Israel

**Keywords:** Working memory, Span task, Modality, Deafness, Blindness, Tactual span

## Abstract

The sensory compensation hypothesis posits that sensory deficits in one modality can lead to enhanced performance of cognitive tasks relying on another, intact modality. Most studies in this area have explored the visual and auditory senses, with inconsistent findings. Meanwhile, the tactile modality has rarely been examined in this context. The present study compared working memory (WM) abilities in the intact senses of individuals with sensory deprivation. Fourteen participants with blindness and 20 with deafness performed a tactile WM task and a verbal or visuospatial WM tasks, respectively. They were compared to 22 age- and education-matched controls who performed all WM tasks. Results showed participants with blindness outperform the other two groups in the tactile WM task and are better than controls in the auditory task. The deafness group outperformed the controls in the visuospatial but not the tactile task. The forward span was longer than the backward span in all modality types and no group by modality interaction was found. Finally, the effect size of differences between blindness and control groups were significantly higher than those of the deafness and control groups' differences. These findings show that blindness and deafness are associated with WM superiority in the intact modality, although not equally. Therefore, the sensory compensation hypothesis in the context of WM is only partially supported as factors, other than deprivation per se may influence performance.

## 1 Introduction

The sensory compensation hypothesis suggests that sensory deprivation in one modality can lead to better than normal performance on cognitive tasks by recruiting the intact modalities (Bell et al., 2019; Braun, 2016). This claim has most often been made with respect to deficits in vision and audition, though research findings are inconsistent (Bell et al., 2019; Megreya and Bindemann, 2017; Merabet and Pascual-Leone, 2010; Pavani and Bottari, 2012).

Among the abilities studied in this context is working memory (WM), or the ability to store and manipulate information for short periods of time (Baddeley, 2012). The original model of WM proposed by Baddeley and Hitch (1974) suggests a differentiation between short term storage of verbal (i.e., phonological loop) and visuospatial (i.e., visuospatial sketchpad; Baddeley, 1986) information. Each modality has its own features that are independent of one another yet are also associated as apparent, for example, in developmental and maturation processes (Bopp and Verhaeghen, 2007; Swanson, 2017), sex differences (Harness et al., 2008), and brain loci (Gogulski et al., 2017).

Later on, Baddeley (2012) addressed additional modalities beyond the known verbal and visuospatial, and suggested that tactile and haptic information are stored in the visuospatial sketchpad. Other authors have described a WM storage component specific to the tactile modality (Cohen et al., 2011) and research has shown tactile WM distinction from other modalities in behavioral (Bliss and Hämäläinen, 2005) and neurological (Kaas et al., 2013) studies, although to a much lesser extent.

Studies have shown inconsistent results regarding WM ability in the intact senses of individuals with blindness or deafness. In relation to blindness and the auditory modality, some indicate better (Dormal et al., 2016; Heled and Oshri, 2021; Pigeon and Marin-Lamellet, 2015; Withagen et al., 2013) and others equal performance compared to controls (Park et al., 2011; Rokem and Ahissar, 2009; Vecchi et al., 2004). However, tactile WM has been shown to be superior and equal to controls (Cohen et al., 2010; Heled and Oshri, 2021; Occelli et al., 2017; Vecchi et al., 2004), but also worse (Aleman et al., 2001; Cornoldi et al., 1991; Vecchi, 1998; Vecchi et al., 2004). Occelli et al. (2017) showed that verbal abilities, including WM, of individuals with congenital blindness were better than intact controls, while in tactile-spatial tasks there was no difference between the groups. They suggested that in blindness there is greater dependence on verbal information than other intact senses, and that this therefore leads to enhanced verbal skills, mainly through practice. Cornoldi and Vecchi (2004) added that lack of vision does not prevent the development of visuospatial WM ability, although it still may be limited compared to controls (Cornoldi et al., 1991). Therefore, blindness might present WM differentiation between modalities, but other factors also contribute to the differences in performance such as task characteristics (e.g., task demand and level of cognitive load) or individual differences (e.g., type of strategic use; Cornoldi and Vecchi, 2004).

Studies on visual WM in individuals with deafness who are skilled in sign language show better performance than intact controls (Ding et al., 2016; Malaia and Wilbur, 2018; Marschark et al., 2013; Moberly et al., 2016; Rudner, 2018), which is mainly explained by sign language skill, with its focus on facial features such as the eyes, eyebrows and mouth in order to communicate, thereby improving visuospatial abilities (Geraci et al., 2008; Lee et al., 2021; Sehyr et al., 2020). Alternatively, others showed equal or inferior visual WM (Koo et al., 2008; Marschark et al., 2016), thus indicating that their superiority is inconclusive, which calls for further exploration of these findings. In the tactile modality, one study showed no difference from controls on a tactile WM task (Heled and Ohayon, 2021) although another showed better performance on a tactile short term memory task (Papagno et al., 2017).

While the sensory compensation hypothesis suggests an advantage in intact modalities, it is unclear whether WM in the tactile modality and other intact modalities are equally influenced in groups with different sensory deficits. A literature search did not reveal studies that compared both storage and manipulation of three different modalities in sensory deprived individuals. Therefore, our aim was to examine individuals with blindness and deafness with respect to WM in the tactile modality as well as in another intact modality (hearing and vision, respectively), while comparing their performance to healthy controls. Consequently, we hoped to better understand modality-specific compensatory mechanisms in WM resulting from sensory deprivation, as a basis for helping professionals in vocational and educational settings to develop more accurate rehabilitation interventions for individuals with blindness and deafness.

## 2 Method

### 2.1 Participants

The study sample comprised 56 Hebrew-speaking adults with no history of developmental, psychiatric, or neurological disorders, who were allocated to three groups according to their sensory status. A blindness group included 14 participants (7 men; mean age = 37.35, SD = 11.6; mean years of education = 15.85, SD = 3.39), 12 with congenital blindness and two who became blind at the age of 2 years (previously presented in Heled and Oshri, 2021). A deafness group included 20 participants (9 men; mean age = 32.55, SD = 11.72; mean years of education = 13.9, SD = 2.73) with congenital hearing loss greater than 80dB (previously presented in Heled and Ohayon, 2021) skilled in sign language. Finally, a control group included 22 adults with no self-reported sensory impairment (10 men; mean age = 34.45, SD = 13.6; mean years of education = 14.45, SD = 2.1) who were matched to the experimental groups with respect to sex, age, and years of education (previously presented in Heled et al., 2020). Individuals with blindness were recruited via the Center for the Blind in Israel. Participants in the deafness and control groups were recruited through employers, social media, and word of mouth. Those who responded to advertisements contacted the examiner and were assessed for compatibility based on self-reported clinical and demographic characteristics. All received the local currency equivalent of 15 USD, apart from 8 control participants who were graduate students at Ariel University and received course credit. The study used data from previous research abovementioned, that was approved by Ariel University ethics committee for the blindness (approval number: AU-EH-20170730) and deafness (approval number: AU-EH-20181204) groups and was not preregistered. All participants gave their consent for filling out a demographic questionnaire and performing the tasks for purpose of data collection.

### 2.2 Instruments

#### 2.2.1 Demographic and clinical questionnaire

Demographic and clinical information was collected using a self-report questionnaire created for the study.

#### 2.2.2 Tactual Span

The deafness and control groups were blindfolded for this task, which aimed at evaluating WM in the tactile modality (Heled et al., 2020). Participants sat in front of the examiner with a table between them on which there was a computer keyboard. Participants placed four fingers of each hand on the upper row keys of the keyboard, and the examiner touched their fingers in a certain order for 1 second each. They were then asked to press the keys with the same fingers touched by the examiner (i.e., a sequence) immediately after their presentation, in the same order (forward stage) or in reverse order (backward stage). The stimuli were composed of three trials per sequence length (right hand, left hand, both hands), starting with 2-finger and reaching up to 9-finger sequences. If participants recalled the entire sequence correctly on at least one trial of the same length, then another stimulus was added and three one stimulus longer sequences were presented. However, if the participant failed to recall all three trials of the same length, the task was stopped. The number of items in the longest sequence recalled correctly in at least one trial served as the dependent variable.

#### 2.2.3 Auditory Span

Only the blindness and control groups performed a computerized Auditory Span task based on the Wechsler digit span task (Wechsler, 1997). Participants were orally presented with a string of numbers by a computer at the rate of 1 second per number, and asked to repeat them immediately after their presentation, in their exact order (forward stage) or in reverse order (backward stage). There were two trials for each sequence length, which was increased by one stimulus until the participant failed to recall two trials of the same length. The number of items in the longest sequence recalled correctly in at least one trial served as the dependent variable.

#### 2.2.4 Visuospatial Span

Only the deafness and control groups performed the Visuospatial Span task, which was a computerized version of the Corsi Block-Tapping task (Corsi, 1972). Nine purple squares were presented on a computer screen in a disorganized array. A sequence of squares changed to yellow, one by one for 1 second each, in a particular order. Participants were asked to point to the squares in the same order (forward stage) or in reverse order (backward stage) immediately after presentation. There were two trials for each sequence length, which was increased by one stimulus until the participant failed to recall two trials of the same length. The number of items in the longest sequence recalled correctly in at least one trial served as the dependent variable.

### 2.3 Procedure

Prior to initializing the experiment, participants signed an informed consent form and were told they are free to discontinue it whenever they want with no implications for them of any kind. Next, they completed the study tasks which were introduced in a counterbalanced order in a single session lasting approximately 1 hour.

### 2.4 Data analysis

First, the groups were compared with respect to age and education using a one-way multivariate analysis of variance (MANOVA) and sex using a chi-square test. Next, in light of the relatively small sample size, the variables were tested for normality and homoscedasticity, to see if parametric tests could be used (Morgan, 2017). Normality was assessed using skewness and kurtosis measures. If the measure divided by the standard error resulted in a score lower than 1.96, the distribution was considered normal (Kim, 2013). Homoscedasticity was measured using Levene's test for equality of variance between the experiment and control groups, while, if violated, Welch's ANOVA test was applied (Morgan, 2017). Then, to test for between-group differences in the Tactual Span, a 2 (Stage: forward/backward) X 3 (Group: blindness/deafness/controls) repeated measures ANOVA was conducted as all participants performed this task. This was followed by a Bonferroni post-hoc test. Next, in order to evaluate differences in sensory deprived groups versus controls in the intact senses (i.e., auditory and visuospatial), two 2 (Stage: forward/backward) X 2 (Group: sensory deprived/controls) repeated measures ANOVA were performed for each sense. In order to determine which WM ability is better in the intact senses of the sensory deprived groups we first extracted Cohen's d value from Group main effects. Then, we compared those effect sizes by calculating Cohen's d variance: Var(d) = (N_1_+N_2_)/(N_1_∗N_2_)+d_2_/(2∗(N_1_+N_2_-_2_)), followed by the Z-scores of the difference between each pair of effect size estimators: Z=(d_1_-d_2_)/$\sqrt{Var\left(d1\right)–Var\left(d2\right)}$ (Hedges, 1981). A Z-score higher than 1.96 was considered significant.

## 3 Results

No between-group differences were found in age and education (*F*(2,55) = 1.11, *p* = .355, η^2^p = .04) or in sex (*χ*^*2*^(55) = .09, *p* = .953). Skewness and kurtosis analyses in each task's stage showed adequate normality values except for one measure in the kurtosis measure. Thus, parametric analyses could be performed (see Table 1). Levene's tests were conducted on all dependent variables, showing non-significant results for all except one comparison: Tactual Span forward *(F* = (2,53) = 1.1, *p* = .338), Auditory Span forward (*F*(1,34) = 1.65, *p* = .207), Visuospatial Span forward (*F*(1,40) = .12, *p* = .721), Tactual Span backward *(F*(2,53) = 1.91, *p* = .158), Auditory Span backward (*F*(1,34) = 6.83, *p* = .013) and Visuospatial Span backward (*F*(1,40) = 1.08, *p* = .304).

**Table 1:** Normality measures of Skewness, Kurtosis and standard error in the dependent variables.

Variable	Skewness	SE	*Kurtosis*	SE
Auditory Span forward	.06	.39	-1.2	.76
Auditory Span backward	-.6	.39	-.31	.76
Tactual Span forward	.18	.32	-.46	.62
Tactual Span backward	-.59	.32	.58	.62
Visuospatial Span forward	-.58	.65	1.47∗	.71
Visuospatial Span backward	.26	.65	-.74	.71

*Note.* SE = standard error, ∗z > 1.96.

**Figure 1:**
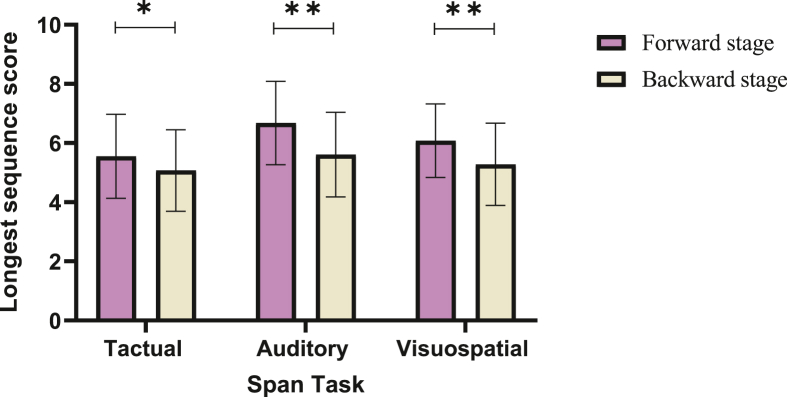
Means and standard deviations of longest sequence span scores by task and stage. Note. ∗*p* < .05, ∗∗*p* < .001.

Analysis of the Tactual Span showed a main effect for stage (*F*(1,53) = 7.53, *p* = .008, η^2^p = .129, revealing that the forward span is longer than the backward span. Another main effect for group was found (*F*(2,53) = 18.5, *p* < .001, η^2^p = .411), and a post-hoc Bonferroni test showed that the participants with blindness performed better than those with deafness (*p* < .001, Cohen's d = 1.72) and control (*p* < .001, Cohen's d = 2.18) groups, which did not differ (*p* = .582, Cohen's d = .36). Finally, no interaction effect for stage (i.e., forward and backward) and group (i.e., blindness, deafness and controls) was found (*F*(2,53) = .53, *p =* .592, η^2^p = .2). Comparing the performance of participants with blindness group and controls in the Auditory Span of both forward and backward stages, showed a main effect for stage (*F*(1,34) = 41.46, *p* < .001, Welch's test = 13.78, *p* < .001, η^2^p = .549). where the forward sequence length was longer than the backward. Group main effect was also significant (*F*(1,34) = 39.32, *p* < .001, Welch's test = 50.31, *p* < .001, η^2^p = .536), where the blindness group performed better than controls. No interaction effect for stage (i.e., forward and backward) and group (i.e., blindness and controls) was found (*F*(1,34) = .21, *p =* .649, η^2^p = .006). The analysis of deafness and control groups revealed a main effect for stage (*F*(1,40) = 13.01, *p* < .001, η^2^p = .246) in the Visuospatial Span, such that the sequence score for the forward stage was longer than the backward stage (see Figure 1 for stage comparison across modalities).

**Figure 2:**
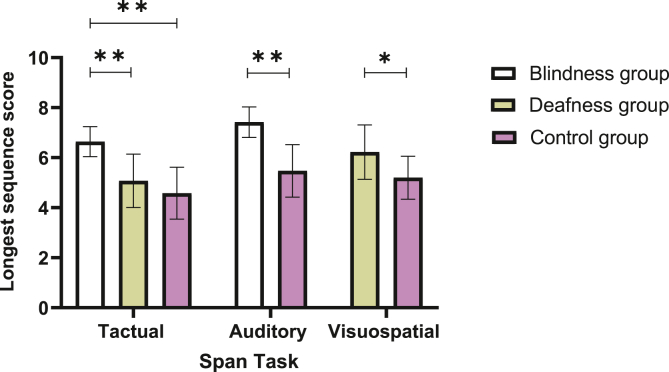
Means and standard deviations of longest sequence span scores by task and group. Note. ∗*p* < .05, ∗∗*p* < .001.

Additionally, a main effect for group was also found (*F*(1,40) = 11.31, *p* = .002, η^2^p = .22), where the deafness group performed better than the controls (see Figure 2 for group comparison across modalities). No interaction effect for stage (i.e., forward and backward) and group (i.e., deafness and controls) was found (*F*(1,40) = .35, *p =* .567, η^2^p = .009).

**Figure 3:**
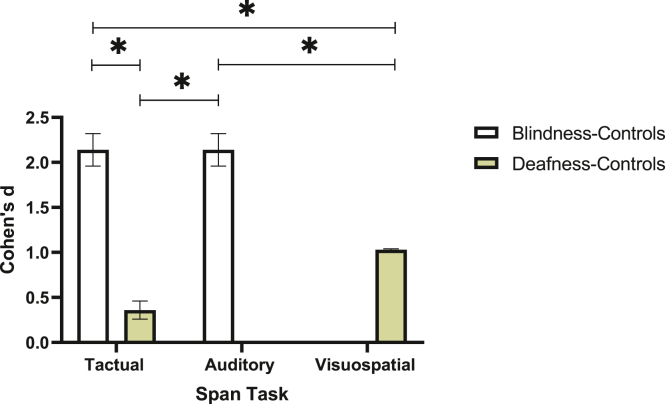
Cohen's d values and standard deviations of group differences by task. Note. ∗*p* < .001.

Finally, we conducted a comparison of Cohen's d effect sizes of the differences between the sensory deprived groups and controls in each intact sense (i.e. visual/auditory and tactile) for the purpose of evaluating WM differences in the intact senses. After extracting Cohen's d and its variance estimates, results showed that the effect size of the blindness-controls comparison in the Tactual Span was significantly higher than that of the deafness-controls comparison in the Visuospatial Span (Z = 2.77, *p* < .001) and the Tactual Span (Z = 3.87, *p* < .001). In addition, the blindness-controls comparison in the Auditory Span was significantly higher than that of the deafness-controls comparison in the Visuospatial Span (Z = 6.13, *p* < .001) and Tactual Span (Z = 8.72, *p* < .001; see Figure 3). We then compared Cohen's d values between the intact modalities in each sensory deprived group. In the deafness group, we found a significant difference between Visuospatial and Tactual Span (Z = 2.22, *p* < .001), while in the blindness group effect sizes were the same in the Tactual and Auditory Spans, indicating no difference between modalities (Z = 0, *p* = n.s).

## 4 Discussion

The current findings showed that the blindness group performed better than the other two groups in the Tactual Span, and better than controls in the Auditory Span. In addition, the deafness group performed similarly to controls in the Tactual Span but better in the Visuospatial Span. The forward span was longer than the backward span in all modalities and no interaction effect was found. Finally, the blindness group exhibited significantly higher effect sizes than the deafness group in their intact senses, and showed no difference between the Tactual and Auditory Spans. The deafness group, on the other hand, performed better on the Visuospatial Span than on the Tactual Span.

Our findings are in accordance with previous reports of superior WM abilities in individuals with blindness, as compared to controls in the auditory (Beauvais et al., 2004; Bliss et al., 2004; Dormal et al., 2016; Withagen et al., 2013) as well as tactile modalities (Cohen et al., 2011; Papagno et al., 2017). This indicates that lack of sensory ability may improve WM performance in the intact senses, presumably as a result of extended use of those senses (Bolognini et al., 2010; Cohen et al., 2010). Occelli et al. (2017) suggested that improvement may be accounted for by developing efficient strategies that enhance performance. However, their suggestion of a differentiation between auditory and tactile-spatial information processing does not coincide with our results as we did not reveal any difference between modalities. This could be a result of using different tasks to tap tactile WM (i.e., Tactual Span versus haptic version of the Corsi block tapping test), and therefore further research is needed.

Exploring WM in the deafness group suggested that the WM superiority in blindness is not consistent in other sensory deprived, because the deafness group outperformed controls only in the visuospatial, but not in the tactile WM task. This implies that sensory deprivation per se does not improve WM abilities in the intact senses, and superiority may be dependent on other factors such as the extent of use in a certain sense (Papagno et al., 2017). Indeed, individuals with blindness appear to use tactile or haptic WM (e.g., Braille reading, navigation) to a far greater extent than do individuals with deafness, and even more so compared to individuals without sensory deprivation. This, in turn, can enhance haptic and tactile WM and help develop strategies to improve WM ability (Cattaneo et al., 2008; Cohen et al., 2010; Picard et al., 2010). Similarly, the differences found between the deafness and control groups might be accounted for by sign language skill, which relies on both visuospatial information processing and language skills, and could therefore potentially contribute to enhancement of visuospatial WM in individuals with deafness (Emmorey et al., 2017; Marschark et al., 2015) alongside other visuospatial cognitive abilities (Boutla et al., 2004; Geraci et al., 2008; Sehyr et al., 2020). This seems not to affect WM in the tactile modality as we found that tactile and visuospatial modalities differ.

Furthermore, effect size comparison showed superiority of the blindness over the deafness group in WM overall, which may indicate that individuals with blindness rely on two modalities (as opposed to only one in individuals with deafness), giving them an advantage in WM function. This suggests that the extent of modality use can explain differences not only between controls and individuals with sensory deprivation but between groups with sensory deprivation in different modalities as well (Heled and Ohayon, 2021; Malaia and Wilbur, 2018; Papagno et al., 2016). It could also lend theoretical support to the domain-specific nature of WM (Cattaneo and Vecchi, 2008), not just of storage but also of manipulation capacity.

Indeed, our findings also showed that trial sequence was longer in the forward compared to the backward stage above and beyond modality type. This supports previous work showing a distinction between the two stages (Farrand and Jones, 1996), thereby indicating that storage of information as tapped by the forward stage requires less cognitive effort than manipulation as tapped by the backward stage. The fact that we found this pattern in all modalities not just strengthens the differentiation between storage and manipulation (Baddeley, 1986), but also shows, following the lack of interaction effects as well, that sensory deprivation influences both these WM components equally. Although research presents a clear distinction between forward and backward spans in the auditory modality but much less so in the visuospatial modality among healthy adults (Donolato et al., 2017; Monaco et al., 2013), our results show that as far as sensory deprivation is concerned the distinction appears in the auditory as well as spatial domain.

However, the findings should be interpreted with caution given several limitations. First, group performance could be accounted for by sign language and Braille skill levels, which could have influenced performance in the Tactual and Visuospatial Spans. Second, use of non-tactile strategies could also have affected Tactual Span performance and it would be useful to test and analyze it in subsequent work. Future studies should include sign language and Braille reading in analyses and also test touch-typing skill influence on performance in the Tactual Span. Additionally, studies could focus on modality-based differences in other cognitive abilities among individuals with sensory deprivation and explore the practical implications of WM on daily functioning.

To summarize, blindness shows superior tactile WM abilities compared to deafness and intact individuals, while also exhibiting better auditory WM compared to controls. Deafness presents better visuospatial but not tactile WM than controls. In addition, sensory deprivation affects WM components equally. Taken together, blindness and deafness may be associated with WM advantage, although sensory damage does not seem to be the sole reason for it. According to this interpretation, the sensory compensation hypothesis is true to the extent of other factors that influence WM performance, and not purely sense damage.

## Declarations

### Author contribution statement

Eyal Heled: Conceived and designed the experiments; Analyzed and interpreted the data; Contributed reagents, materials, analysis tools or data; Wrote the paper.

Or Oshri & Maayan Ohayon: Performed the experiments; Analyzed and interpreted the data.

### Funding statement

This research did not receive any specific grant from funding agencies in the public, commercial, or not-for-profit sectors.

### Data availability statement

Data will be made available on request.

### Declaration of interest’s statement

The authors declare no conflict of interest.

### Additional information

No additional information is available for this paper.

## Appendix A Supplementary data

Demographic questionnaire Demographic questionnaire
