# Supplementary material for: Working memory in intact modalities among individuals with sensory deprivation
Source: Heliyon. 2022 May 29;8(6):e09558. doi: 10.1016/j.heliyon.2022.e09558 (PMC9189883; doi:10.1016/j.heliyon.2022.e09558)
Supplement: Demographic questionnaire [file mmc1.docx]

**Demographic questionnaire (Deafness research)**

Participant number:

sex: Man/Woman

age:

Number of education years:

Dominant hand:

Congenital deafness: Yes/No. If no, Age of deafness:

Decibels hearing loss:

Cause of deafness:

Cochlear Implant: none/right/left/both

Language:

Native language (Indicate if sign language was a first language):

Speaks sign language: Yes/No. If yes, sign language acquisition age:

Proper speech: Yes/No

Any psychiatric diagnoses past or present? Yes/No. If yes, please state type _____________.

Any neurological damage – congenital or acquired (e.g., attention, learning disability, brin injury etc.)? Yes/No. If yes, please state type _____________.

Any sensory or fine motor problem or using a computer mouse? Yes/No.

**Demographic questionnaire (Blindness research)**

Participant number:

sex: Man/Woman

age:

Number of education years:

Dominant hand:

Congenital blindness: Yes/No. If no, Age of blindness:

Native language:

Any psychiatric diagnoses past or present? Yes/No. If yes, please state type _____________.

Any neurological impairment – congenital or acquired (e.g., attention, learning disability, brin injury etc.)? Yes/No. If yes, please state type _____________.

Any sensory or fine motor problem? Yes/No.
